# Diet for the prevention and treatment of Parkinson’s disease

**DOI:** 10.3389/fnut.2025.1587246

**Published:** 2025-12-17

**Authors:** Wenzhi Chen, Ting Fan, Renshi Xu

**Affiliations:** 1 Department of Neurology, Jiangxi Provincial People’s Hospital, The Clinical College of Nanchang Medical College, The First Affiliated Hospital of Nanchang Medical College, Xiangya Hospital of Center South University, Jiangxi Hospital and National Regional Center for Neurological Disease, Nanchang, Jiangxi, China; 2 School of Clinical Medicine, Jiangxi University of Traditional Chinese Medicine, Nanchang, China

**Keywords:** Parkinson’s disease, polyphenols, fatty acids, vitamins, dietary pattern

## Abstract

Parkinson’s disease (PD) is a common degenerative disease of the central nervous system with a variety of non-motor symptoms, in addition to the typical motor symptoms, which seriously affect the physical and mental health of patients, as well as their quality of life. All current treatment measures for PD only ameliorate and alleviate partial symptoms and signs in the early and middle stages. To date, no medicine or surgery can prevent PD progression. The search for preventive measures to treat and prevent PD is currently a research priority. Based on current related studies and investigations, diet interventions have now been found to play important roles in the treatment and prevention of PD. This article reviews recent research advances in diet in terms of polyphenols, fatty acids, vitamins, and dietary patterns for the prevention and treatment of PD.

## 1 Introduction

Parkinson’s disease (PD) is a neurodegenerative disease commonly present in middle-aged and elderly populations, with insidious onset and progressive exacerbation. Its clinical manifestations include motor symptoms, such as bradykinesia, resting tremor, myotonia, and postural balance disorders, and non-motor symptoms, such as hyposmia, sleep disturbance, constipation, and mental and cognitive impairment (1). The main pathological features of PD are the absence of dopaminergic neurons in the dense substantia nigra and accumulation of toxic Lewy bodies in the cytoplasm of neurons (1–3).

At present, the treatment of PD only ameliorates and alleviates the partial symptoms and signs at the early and mid-stages, cannot prevent and squelch the pathological lesions of PD, and cannot reverse and stop the progression of PD (2). The current major treatment of PD supplies the deficiency of dopaminergic neurotransmitters and/or reduces the degradation of dopaminergic neurotransmitters in the synaptic gap of the striatum, or the use of dopaminergic agonists to model dopaminergic neurotransmitters to stimulate the dopaminergic receptor to relieve the symptoms and signs, mainly consisting of compound levodopa agents (dopaminergic neurotransmitter supplemental drugs), monoamine oxidase B and catecholamine O-methyltransferase (dopaminergic neurotransmitter degradation inhibiting drugs), and dopaminergic receptor agonists (directly stimulating dopaminergic receptor drugs) (4).

In addition to medicinal treatment for PD, surgery such as deep brain stimulation also partially eliminates the symptoms and signs of PD (5). Despite the availability of numerous therapeutic measures, none can prevent or cure PD (3). Furthermore, with the aging of the global population, the rising prevalence of PD worldwide poses a serious threat to human health and a significant socioeconomic burden in many countries and regions (3, 6).

Therefore, finding new ways to prevent and treat PD remains a top priority in PD research and investigation. Based on the current progression of PD treatment and prevention, diet is receiving increasing attention from many researchers and is thought to be another important measure for the treatment and prevention of PD besides pharmacy and surgery (7). Research and investigation evidence indicates that diet is considered a potential environmental factor in the development or progression of PD, and there is growing evidence that diet may play an important role in PD (8). This article briefly reviews the role of diet and dietary patterns in the prevention and treatment of PD.

## 2 Polyphenols

**Figure 1:**
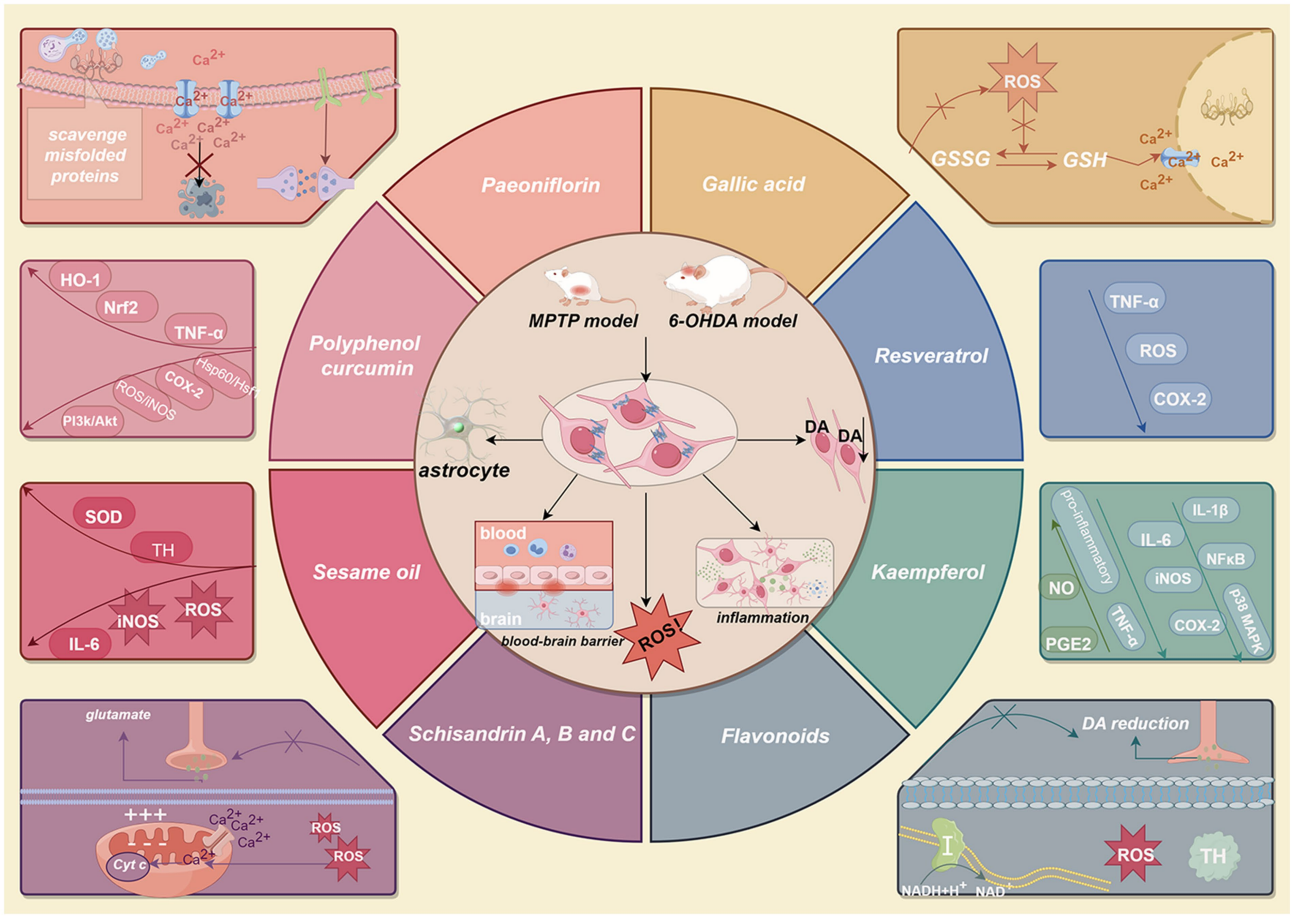
The potential effects of a polyphenol diet on PD patients. In the MPTP and 6-OHDA rodent (mouse and rat) models, MPTP and 6-OHDA induce a α-synuclein increase, activates astrocytes and destructs blood–brain barrier, which cause inflammation, oxidative stress and decrease dopaminergic neurons. This figure lists the various potential functions of 10 important polyphenols (paeoniflorin, gallic acid, flavonoids, kaempferol, resveratrol, schisandrin A, B and C, sesame oil, polyphenol curcumin) in PD, such as antioxidant, anti-inflammatory, neurogenesis, neuroprotective, and elimination of misfolded proteins in PD. Created with Figdraw (www.figdraw.com). MPTP, 1-methyl-4-phenyl-1,2,3,6-tetrahydropyridine; 6-OHDA, 6-hydroxydopamine; PD, Parkinson’s disease.

The role of polyphenol-rich diets in PD is an active area of research, although conclusive clinical evidence remains limited (9). Preclinical studies have shown that natural polyphenols can mitigate key pathogenic processes in PD (10). For example, the neurotoxin models of PD (using MPTP or 6-OHDA) recapitulate features of the disease—elevated α-synuclein, astrocyte activation, blood–brain barrier disruption, inflammation, oxidative stress, and dopaminergic neuron loss—and these models have demonstrated the neuroprotective potential of various dietary polyphenols (10). Epidemiological data also suggest that polyphenol-rich dietary patterns (e.g., Mediterranean or Asian diets) are associated with reduced neurodegeneration and lower incidence of PD (11). Polyphenols exert their effects by targeting multiple pathways implicated in PD, including neuroinflammation, oxidative stress (reactive oxygen species, ROS), and protein aggregation, thereby potentially protecting vulnerable neurons (12) (Figure 1). Many polyphenols can directly scavenge free radicals and also induce endogenous antioxidant defenses (e.g., upregulating glutathione, catalase, and superoxide dismutase), which helps restore redox balance in neuronal cells (10).

Polyphenols are a diverse group of plant secondary metabolites broadly categorized into flavonoids and non-flavonoids. The non-flavonoid polyphenols include classes such as phenolic acids, stilbenes, and lignans (as well as others like xanthones and tannins). In the context of PD, a variety of compounds from each class have shown beneficial effects (13). Below, we summarize four major categories of polyphenols—flavonoids, phenolic acids, stilbenes, and lignans—highlighting representative compounds and their potential roles in PD prevention or therapy.

### 2.1 Flavonoids

Flavonoids are the largest family of polyphenols, comprising over 4,000 compounds and accounting for roughly 60% of dietary polyphenols (14). They share a common phenylbenzopyrone structure and are subdivided into various subclasses, including flavonols, flavones, flavanones, flavan-3-ols (catechins), anthocyanins, isoflavones, and others (14). A higher intake of flavonoids—particularly anthocyanins found in berries, grapes, and red wine—has been associated with a lower risk of developing PD in human cohort studies (9). Notably, one large prospective study showed that men in the highest quintile of flavonoid consumption had a significantly reduced PD risk compared to those with the lowest intake (with anthocyanin-rich foods being especially protective) (15). There is also evidence that flavonoid consumption may benefit those already diagnosed with PD: for example, diets rich in berries, tea, and other flavonoid sources have been linked to slower cognitive decline and even lower mortality in PD patients (16). These epidemiological findings underscore the potential of flavonoids as neuroprotective agents in the context of PD.

Mechanistic studies in cell and animal models of PD have demonstrated multiple neuroprotective actions of flavonoids (17). In toxin-based models (MPTP or 6-OHDA), flavonoids prevent dopaminergic neuron dysfunction by preserving dopamine levels, reducing oxidative damage, and maintaining tyrosine hydroxylase (TH) protein expression (18). Many flavonoids also counteract neuroinflammation and apoptosis. For instance, kaempferol (a flavonol abundant in foods like saffron, apples, and leafy greens) protects against α-synuclein toxicity: in PD models it increases neuronal viability, decreases misfolded α-synuclein aggregates, prevents TH-positive neuron loss, and attenuates astrocyte activation (as indicated by reduced glial fibrillary acidic protein, GFAP) (19). The flavonols quercetin and fisetin (found in fruits and vegetables such as apples, berries, and persimmons) similarly promote neuronal survival and anti-inflammatory effects (20, 21). Quercetin and fisetin treatment in MPTP-lesioned mice or cell cultures increases cell viability and inhibits apoptosis, accompanied by lower levels of proinflammatory cytokines like tumor necrosis factor-α (TNF-α) and interleukin-6 (IL-6) (22). Fisetin has been shown to reduce nitric oxide (NO) and prostaglandin E₂ production by inhibiting the inducible nitric oxide synthase (iNOS), cyclooxygenase-2 (COX-2), and IL-1β pathways (23). Both quercetin and fisetin are notable for their ability to stabilize cellular glutathione levels during oxidative stress, thereby preserving the antioxidant capacity of dopaminergic neurons (24). At the molecular level, these flavonols attenuate the activation of NF-κB and p38 MAPK inflammatory signaling; fisetin in particular blocks the degradation of IκB (the inhibitor of NF-κB), which prevents the nuclear translocation of NF-κB and the downstream inflammatory cascade (25). Beyond these examples, numerous other flavonoids confer neuroprotection via antioxidant response pathways (9). Many flavonoids (including hesperetin from citrus, farrerol from rhododendron, and the flavones apigenin and luteolin from chamomile and celery) activate the Keap1–Nrf2–ARE pathway, which leads to upregulation of cytoprotective enzymes such as heme oxygenase-1 and superoxide dismutase (17, 26). Through Nrf2 activation, these compounds enhance the cell’s intrinsic defense against oxidative injury in PD models. Consistently, apigenin and luteolin treatment in MPTP mice was shown to preserve TH levels and significantly reduce striatal TNF-α, thereby dampening neuroinflammation *in vivo* (27). Certain flavonoids can even promote neuronal recovery: fisetin, quercetin, luteolin, and others have been observed to induce neurite outgrowth and synaptic marker expression, suggesting a capacity to support neural regeneration, although this requires continuous presence of the compounds and appears dose-dependent (9).

It is worth noting that flavonoids encompass many compounds beyond those detailed here. Other subclasses—for example, the flavan-3-ols (catechins found in green tea) and isoflavones (soy-derived phytoestrogens)—also exhibit neuroprotective activities in experimental models of PD (28). Epigallocatechin gallate (EGCG), a catechin from green tea, can reduce α-synuclein aggregation toxicity and oxidative damage in PD models, and isoflavones like biochanin A and genistein have shown anti-inflammatory and neurotrophic effects in dopaminergic cells (29). These compounds are not discussed in detail in this section, but they further highlight the broad spectrum of flavonoid neuroprotectants being explored for PD. Overall, flavonoids act on multiple pathogenic targets—oxidative stress, inflammation, mitochondrial dysfunction, and protein aggregation—to confer neuroprotection, supporting epidemiological observations that flavonoid-rich diets may help prevent or slow the progression of PD.

### 2.2 Phenolic acids

Phenolic acids are a class of polyphenols characterized by one aromatic ring with one or more hydroxyl groups and an attached carboxylic acid (30). They are commonly divided into hydroxybenzoic acids (e.g., gallic, p-hydroxybenzoic acids) and hydroxycinnamic acids (e.g., caffeic, ferulic, p-coumaric acids) (31). These compounds are widespread in plant-based foods (for instance, gallic acid in berries and tea, caffeic acid in coffee, ferulic acid in whole grains) (32). Compared to flavonoids, relatively few studies have focused on phenolic acids in PD, but emerging evidence suggests certain phenolic acids can exert neuroprotective effects (33). Two notable examples are paeoniflorin and gallic acid, which have been investigated for their anti-Parkinson properties.

Paeoniflorin is a monoterpene glycoside (sometimes categorized as a phenolic glycoside) abundant in the root of *Paeonia lactiflora* (white peony) (34). In PD research, paeoniflorin has shown promising neuroprotective activity. Animal studies indicate that paeoniflorin administration can ameliorate PD-like motor deficits and prevent the loss of dopaminergic neurons in both MPTP mouse and 6-OHDA rat models (35, 36). One mechanism underlying these benefits is the activation of adenosine A₁ receptors by paeoniflorin: A₁ receptor stimulation has neuroprotective effects, and paeoniflorin’s ability to activate this pathway is linked to reduced neuroinflammation and improved dopamine neurotransmission in toxin-induced PD models (37). In addition, paeoniflorin engages multiple intracellular targets to promote neuronal survival. It has been shown to enhance the autophagy–lysosome pathway responsible for clearing misfolded proteins like α-synuclein (38). By facilitating the removal of protein aggregates, paeoniflorin may reduce one source of toxicity in PD. Concordantly, *in vitro* experiments demonstrate that paeoniflorin prevents the buildup of α-synuclein in dopaminergic cells exposed to MPP^+ and alleviates the associated cytotoxicity (38). Paeoniflorin also helps maintain cellular homeostasis by limiting pathological calcium influx and oxidative stress-induced damage (38). In MPTP-treated neuronal cultures, paeoniflorin restored cell viability, normalized elevated intracellular Ca^2+^, and decreased apoptosis markers relative to untreated controls (38). These multifaceted actions (anti-inflammatory, pro-autophagic, anti-oxidative) make paeoniflorin a compelling phenolic candidate for further PD research.

Gallic acid (3,4,5-trihydroxybenzoic acid) is another phenolic acid with demonstrated neuroprotective effects in PD models. Gallic acid is found in various fruits and plant products (such as berries, tea, nuts, and red wine) either in free form or as part of tannins and other polyphenols (32). Although gallic acid itself is a simple molecule, its antioxidant potency is well known. In cellular models of PD, gallic acid has been shown to improve neuronal survival under toxin exposure. For example, in 6-OHDA–treated dopaminergic cell cultures, gallic acid significantly increased cell viability and reduced markers of apoptosis compared to untreated cells (39). Interestingly, certain gallic acid derivatives appear even more effective: metabolites such as propyl gallate, methyl gallate, and other alkyl gallate esters exhibited stronger neuroprotective effects than gallic acid in some PD models (9). In one comparative study, n-propyl gallate provided the most robust protection across multiple readouts of neuroprotection, outperforming gallic acid and other analogues (40). Mechanistically, gallic acid and its esters combat the oxidative and inflammatory damage in PD through several pathways. They potently attenuate ROS accumulation and oxidative stress in dopaminergic cells, thereby preventing the depletion of endogenous antioxidants. Treatment with gallic acid has been shown to lower levels of oxidized glutathione (GSSG) while increasing reduced glutathione (GSH), reflecting a restoration of the cellular redox balance (40). Gallic acid also helps stabilize intracellular calcium levels; by reducing aberrant Ca^2+^ influx, it can prevent calcium-mediated excitotoxicity and mitochondrial dysfunction in neurons (40). Furthermore, gallic acid has been reported to inhibit the aggregation of α-synuclein and promote the clearance of this toxic protein from cells (41). Together, these effects—antioxidative, anti-aggregative, and calcium-stabilizing—enable gallic acid to reduce dopaminergic cell death in PD models. Gallic acid is also notable for its bioavailability; among dietary polyphenols, it is relatively well absorbed and can cross the blood–brain barrier (42), making it a particularly interesting molecule for potential neuroprotective therapy. Ongoing studies are examining gallic acid derivatives and formulation strategies to enhance its neuroprotective efficacy.

In summary, while research on phenolic acids in PD is still emerging, compounds like paeoniflorin and gallic acid exemplify how these polyphenols can intervene in PD pathogenesis by modulating neurotransmitter systems, enhancing protein clearance mechanisms, and protecting neurons from oxidative/inflammatory injury. Their positive outcomes in preclinical models warrant further investigation, including whether these phenolic acids (or optimized derivatives) can be utilized in dietary interventions or developed into neuroprotective drugs for PD.

### 2.3 Stilbenes

Stilbenes are a relatively small class of polyphenols characterized by a 1,2-diphenylethylene structure. They are produced by plants often as phytoalexins (defense compounds in response to stress or infection) (14). Dietary intake of stilbenes is generally low, as only certain foods like grapes, berries, and peanuts contain significant amounts. The most extensively studied stilbene is resveratrol (trans-3,5,4′-trihydroxystilbene), which has attracted intense interest for its neuroprotective and longevity-enhancing properties. Resveratrol is found in grapes, red wine, blueberries, and other fruits, and numerous studies have explored its beneficial effects in models of neurodegenerative diseases, including PD (43).

Resveratrol has demonstrated robust neuroprotective effects in a variety of PD models. In rodent models of PD, resveratrol administration (either via diet or intravenous injection) significantly protects against motor deficits and dopaminergic neurodegeneration induced by MPTP or 6-OHDA (44). Treated mice show preservation of striatal dopamine levels and TH-positive neuron counts in the substantia nigra compared to untreated toxin-lesioned controls (45). Resveratrol also mitigates the glial activation and oxidative damage caused by these neurotoxins (46). For example, in MPTP-treated mice, resveratrol prevented the increase in malondialdehyde and other indicators of lipid peroxidation, and maintained normal antioxidant enzyme activity in the brain (15). In parallel, resveratrol markedly reduces neuroinflammation in toxin models: studies report that resveratrol-treated PD model animals have lower levels of pro-inflammatory mediators such as COX-2, TNF-α, and IL-1β in the nigrostriatal system, as well as reduced ROS accumulation (47). These anti-inflammatory and antioxidant effects translate into histological protection (less neuronal loss) and functional improvements in motor behavior.

At the molecular level, resveratrol targets multiple cell signaling pathways to exert its neuroprotective actions. It is a potent free-radical scavenger and can directly neutralize ROS, thereby protecting neurons from oxidative stress-induced apoptosis. Resveratrol also inhibits lipid peroxidation in neuronal membranes, preserving cell integrity under oxidative challenge (48). In terms of anti-inflammatory signaling, resveratrol has been shown to interfere with the NF-κB pathway. In dopaminergic cell models, resveratrol treatment increases IκBα levels (the inhibitory subunit that sequesters NF-κB) and correspondingly lowers nuclear translocation of the NF-κB p65 subunit (49). This leads to a downregulation of NF-κB–dependent inflammatory genes, providing a mechanistic basis for resveratrol’s observed reduction of cytokine production in PD models (49). Resveratrol may additionally activate the cell’s endogenous antioxidant responses via the Nrf2 pathway: there is evidence that resveratrol can promote Nrf2 release from Keap1 and its translocation to the nucleus, where Nrf2 induces the expression of detoxifying and antioxidant enzymes (50). This Nrf2-mediated response further boosts the neuronal defense against oxidative injury. Another important mechanism of resveratrol is the activation of mitochondrial biogenesis and function. Resveratrol can activate AMP-activated protein kinase (AMPK) and the downstream peroxisome proliferator-activated receptor-γ coactivator 1α (PGC-1α), which together enhance mitochondrial health and energy metabolism (51). Improved mitochondrial function helps neurons resist energy deficits and apoptotic signals in PD models. Consistent with these cellular findings, resveratrol has shown neuroprotective efficacy across different model systems and species. In Drosophila models of PD, dietary resveratrol increased survival rates and improved locomotor function while reducing oxidative markers in the flies’ brains (52). Given its multi-targeted actions, resveratrol is often highlighted as a candidate for complementary therapy in neurodegenerative diseases. In fact, some researchers have proposed that resveratrol could be combined with other polyphenols for synergistic effects, as it might work in different cellular compartments than, for example, certain flavonoids (9). Overall, the evidence to date suggests that resveratrol can counteract PD pathology by protecting dopaminergic neurons from oxidative and inflammatory injury and by modulating signaling pathways involved in cell survival. Its favorable effects in preclinical PD models (including improvements in motor coordination and neuronal survival) support the idea that resveratrol or resveratrol-rich diets could be beneficial in PD, although clinical studies are needed to confirm its efficacy and optimal dosing. Resveratrol’s prominence among stilbenes exemplifies how even minor components of the human diet can have outsized effects on neural health.

### 2.4 Lignans

Lignans are a class of polyphenols formed by the coupling of two phenylpropanoid units. They are commonly found in high-fiber plant foods such as whole grains, flaxseed, sesame seeds, nuts, and berries (53). In the diet, lignans often occur as complex precursors (like secoisolariciresinol diglucoside in flax) that are metabolized by gut microbiota into bioactive forms (54). Several lignans have shown neuroprotective properties in experimental models of PD. Key examples include schisandrins (from *Schisandra chinensis* fruit), 7-hydroxymatairesinol (found in coniferous trees and in trace amounts in foods like strawberries and sesame), and sesame seed lignans such as sesamin, sesamol, and sesaminol (55–57).

Schisandrin A, B, and C—bioactive lignans from Schisandra berries—have demonstrated protective effects on neurons in models relevant to PD (55). Schisandra extracts have long been used in traditional medicine, and modern studies indicate that schisandrins can shield neurons from excitotoxic and oxidative damage. (55) In primary neuronal cultures, pretreatment with schisandrin significantly attenuated glutamate-induced excitotoxicity, a process implicated in PD progression (58). Schisandrin-treated cells showed lower ROS levels and preservation of mitochondrial membrane potential even after glutamate exposure, compared to untreated cells (58). By reducing excessive Ca^2+^ influx (a trigger of cell death) and preventing the release of pro-apoptotic factors like cytochrome c from mitochondria, schisandrins effectively decreased apoptotic cell death in this model (58). Notably, schisandrin maintained intracellular glutathione (GSH) levels and prevented the depletion of caspase-acting factors that glutamate toxicity would normally cause (58). These findings suggest schisandrins bolster the intrinsic antioxidant and anti-apoptotic defenses of neurons. In *in vivo* PD models, schisandrin A has shown similar beneficial effects. MPTP-treated mice that received schisandrin A had higher levels of TH expression in the striatum, indicating protection of dopaminergic nerve terminals (59). Schisandrin A also modulated autophagy and inflammation in these models: it inhibited excessive oxidative and inflammatory responses (for example, by reducing markers of lipid peroxidation and suppressing microglial activation) and helped normalize autophagy-related proteins that are disrupted in PD (59). In particular, MPTP intoxication typically downregulates key autophagy regulators (LC3-II, beclin-1, parkin, PINK1), but schisandrin A treatment partially preserved the levels of these proteins, implying that it enhances autophagic clearance of toxic aggregates (59, 60). Schisandrin’s anti-inflammatory action in PD models is evidenced by decreased proinflammatory cytokine production; although reductions in TNF-α and IL-1β were modest in one study, the overall trend supports an anti-inflammatory effect (59). In summary, schisandrin lignans appear to protect dopaminergic neurons by a combination of antioxidant, anti-excitotoxic, anti-inflammatory, and pro-autophagic mechanisms, making them interesting candidates for further neuroprotective research.

7-Hydroxymatairesinol (7-HMR) is a lignan originally derived from the knotwood of spruce trees, and it is metabolically related to the mammalian lignan enterolactone (61). Recent studies have explored 7-HMR in neurodegeneration, including PD. In a rat model of PD induced by 6-OHDA, 7-HMR treatment did not significantly prevent the loss of nigral dopaminergic neurons, but it produced notable anti-inflammatory effects in the brain (56). Treated rats showed reduced activation of microglia and astrocytes in the substantia nigra and striatum, as evidenced by lower expression of glial markers compared to untreated 6-OHDA controls (56). 7-HMR also lowered the levels of proinflammatory mediators: brain tissue analyses revealed decreases in TNF-α and iNOS (inducible nitric oxide synthase) with 7-HMR therapy (62). The mechanism of 7-HMR’s anti-inflammatory action may relate to its structural similarity to estrogen; some researchers speculate that 7-HMR, like other plant lignans, can act as a phytoestrogen and thereby modulate inflammatory pathways linked to hormonal signaling (63). Estrogenic compounds are known to have neuroimmune effects (64), and 7-HMR’s inflammation-reducing property in PD models might be partially due to such activity. While 7-HMR alone did not rescue dopaminergic cell loss in the acute toxin model (56), its ability to dampen neuroinflammation could be beneficial in a chronic setting or in combination with other therapies that promote neuron survival. This lignan’s profile suggests it primarily aids in controlling the neuroinflammatory environment in PD, which is an important aspect of slowing neurodegeneration.

Lignans from sesame (*Sesamum indicum*) have also drawn attention for neuroprotection. Sesamin, sesamol, and sesaminol are phenolic compounds present in sesame seeds and oil, known for antioxidant and anti-inflammatory properties (65). In MPTP mouse models of PD, sesame lignans showed protective effects on dopaminergic neurons. Administration of sesamin prior to MPTP exposure significantly reduced oxidative stress in the mouse brain: it suppressed the production of ROS and prevented the induction of iNOS and IL-6 in activated microglial cells (66). These changes corresponded with lower oxidative damage and inflammatory burden in the nigrostriatal pathway. Furthermore, sesamin-treated mice maintained higher levels of endogenous antioxidants – for example, superoxide dismutase (SOD) activity was increased relative to untreated MPTP mice, and striatal TH levels were better preserved, indicating reduced dopaminergic neuron damage (66). Intriguingly, sesamin also modulated other antioxidant enzymes: MPTP typically causes a compensatory rise in catalase (an antioxidant enzyme) due to oxidative stress, but sesamin pretreatment blunted this excessive catalase increase (67). This suggests that sesamin directly quenched ROS and alleviated oxidative burden, making an extreme upregulation of catalase unnecessary. Similarly, sesamol and sesaminol have shown neuroprotective effects in related models, often attributed to their ability to cross the blood–brain barrier and act as free radical scavengers in the brain (68). Collectively, the sesame lignans exhibit a capacity to reduce neuroinflammation and oxidative injury in the PD context, thereby protecting neuronal integrity.

Curcumin, while not a lignan, is another natural polyphenol often discussed alongside these compounds due to its broad neuroprotective profile. Curcumin is a diferuloylmethane derived from turmeric (*Curcuma longa*) and is sometimes referred to as the “golden spice” polyphenol (69). It does not fit neatly into the above polyphenol subclasses (it is classified as a curcuminoid), but its relevance to PD warrants mention. Curcumin exerts multiple pharmacological effects that target PD pathogenesis. It is a powerful antioxidant and anti-inflammatory agent, capable of protecting neurons from a range of insults. Curcumin can directly neutralize ROS and reactive nitrogen species, reducing oxidative damage to lipids, proteins, and DNA in dopaminergic cells (70). It also chelates metal ions (like iron and copper) that contribute to radical generation, further mitigating oxidative stress (71). On the inflammatory front, curcumin inhibits microglial activation and downregulates the expression of inflammatory enzymes and cytokines—for instance, it suppresses iNOS and COX-2 induction and lowers levels of TNF-α and other interleukins in activated glial cells (72). In various PD models (cell culture and animal), curcumin treatment has been shown to reduce markers of neuroinflammation concomitant with preserving dopaminergic neuron function (73). Another critical action of curcumin is its interference with pathogenic protein aggregation. Curcumin can bind to misfolded protein oligomers; in models of synucleinopathy, it inhibits the oligomerization of α-synuclein and promotes the clearance of existing aggregates (74). It also upregulates molecular chaperones and components of the proteostasis network, such as heat shock proteins (HSP60, HSP70) and autophagy-related proteins, facilitating the removal of toxic protein species (75). Additionally, curcumin has been noted to stimulate neurotrophic pathways and neurogenesis. It can activate the phosphoinositide 3-kinase/Akt (PI3K/Akt) signaling pathway, enhancing cell survival signals and contributing to the resilience of neurons under stress (76). Curcumin is often described as a hormetic compound because it induces an adaptive stress response: it mildly stresses cells in ways that activate Nrf2 and other transcription factors, leading to increased production of antioxidant and cytoprotective proteins (e.g., heme oxygenase-1) that precondition cells against subsequent injuries (77, 78). Notably, despite being a large polyphenol, curcumin is lipophilic and can cross the blood–brain barrier at sufficient concentrations to exert effects in the CNS (79). Its multi-targeted actions in models of PD, AD, and other neurodegenerative disorders highlight curcumin’s potential as a neuroprotective nutraceutical. In PD specifically, curcumin has improved motor behavior and dopaminergic neuron survival in some rodent studies, and it continues to be investigated in preclinical and early clinical settings (such as trials of curcumin formulations or analogues with improved bioavailability) (80). Given its safety profile and broad range of beneficial effects (antioxidant, anti-inflammatory, anti-aggregation, and neurogenic), curcumin stands out as a promising adjunctive treatment for PD.

In conclusion, a wide array of polyphenols – spanning flavonoids, phenolic acids, stilbenes, lignans, and other categories – have shown protective effects against the processes that drive PD neurodegeneration. These compounds commonly reduce oxidative stress and inflammation, enhance the cellular mechanisms for damaged protein clearance, and support neuronal survival and function. Accumulating epidemiological and preclinical evidence strongly suggests that long-term consumption of polyphenol-rich foods could help prevent or slow the progression of PD (81). Indeed, several studies have found that adherence to polyphenol-rich diets is associated with a lower risk of developing PD and with better clinical outcomes in PD patients (9). While more clinical trials are needed, the current data indicate that polyphenol compounds hold promise as dietary interventions or lead compounds for novel therapeutics in PD. Embracing a diet high in fruits, vegetables, teas, and other polyphenol sources may therefore confer neuroprotective benefits, potentially improving the quality of life and outcomes for individuals at risk of PD or living with the disease.

## 3 Vitamins

### 3.1 Vitamin D

Vitamin D (VD) is a fat-soluble vitamin consisting mainly of VD2 (ergocalciferol) and VD3 (cholecalciferol). VD3 is synthesized endogenously primarily through sunlight or UV exposure of the skin and can also be obtained from animal foods, whereas VD2 can only be obtained from plant foods or supplements. VD obtained by sunlight exposure or through diet is converted to the main circulating form of VD, 25(OH)D, by 25 hydroxylase. 25(OH)D is biologically inactive and must be converted to its active form, 1,25(OH)2D3, by 1α-hydroxylase in the kidneys (82). VD plays an important role in cell proliferation and differentiation, neurotrophic regulation, neuroprotection, neurotransmission, immune regulation and neuroplasticity (83, 84).

In recent years, the roles of VD in the nervous system have been continuously discovered. The brain substantia nigra in PD has a high level of expression of the VD metabolizing enzyme and 1α hydroxylase, and VD can stimulate the expression of tyrosine hydroxylase, a key enzyme of dopamine synthesis in PD, which affects the production and turnover of dopamine, therefore, the role of VD in the development process of PD has received increasing attention (85, 86). In a total of 20 studies (14 observational studies, 1 intervention study, and 5 rodent studies) for the systematic evaluation and analysis of VD and PD, it was reported in a meta-analysis of six observational studies reporting differences in VD levels that mean serum 25(OH)D levels in PD patients were lower than those in healthy controls, five studies in animal models of PD showed that supplemental VD intake in rodents significantly increased the concentrations of tyrosine hydroxylase, promoted the conversion of dopamine neurotransmitter, significantly decreased oxidative damage to the substantia nigra, and significantly increased dopamine levels and neuronal survival in the substantia nigra (87). However, the high prevalence of VD deficiency in PD patients could also be the result of reduced outdoor activity owing to activity restriction, and the possibility that gastrointestinal dysfunction (a non-motor PD symptom) leads to lower VD2 levels in PD patients cannot be completely ruled out.

A study using liquid chromatography–tandem mass spectrometry to determine plasma levels of total 25(OH)D, 25(OH)D2, and 25(OH)D3 in PD patients and normal subjects found that the risk of developing PD was significantly increased with plasma total 25(OH)D <30 ng/mL. Relative to the highest quartile, the lowest quartiles of VD2 and VD3 concentrations were both significantly negatively associated with the risk of developing PD. This suggests that VD2 deficiency may also contribute to the development of PD (88). Several studies have also found that both VD supplementation and outdoor work are associated with a significantly lower risk of PD (89). In addition, in a randomized double-blind case–control study on whether VD3 supplementation inhibited the progression of PD, VD3 significantly prevented the deterioration of the Hoehn and Yahr scale in patients with PD compared to placebo controls (90).

**Figure 2:**
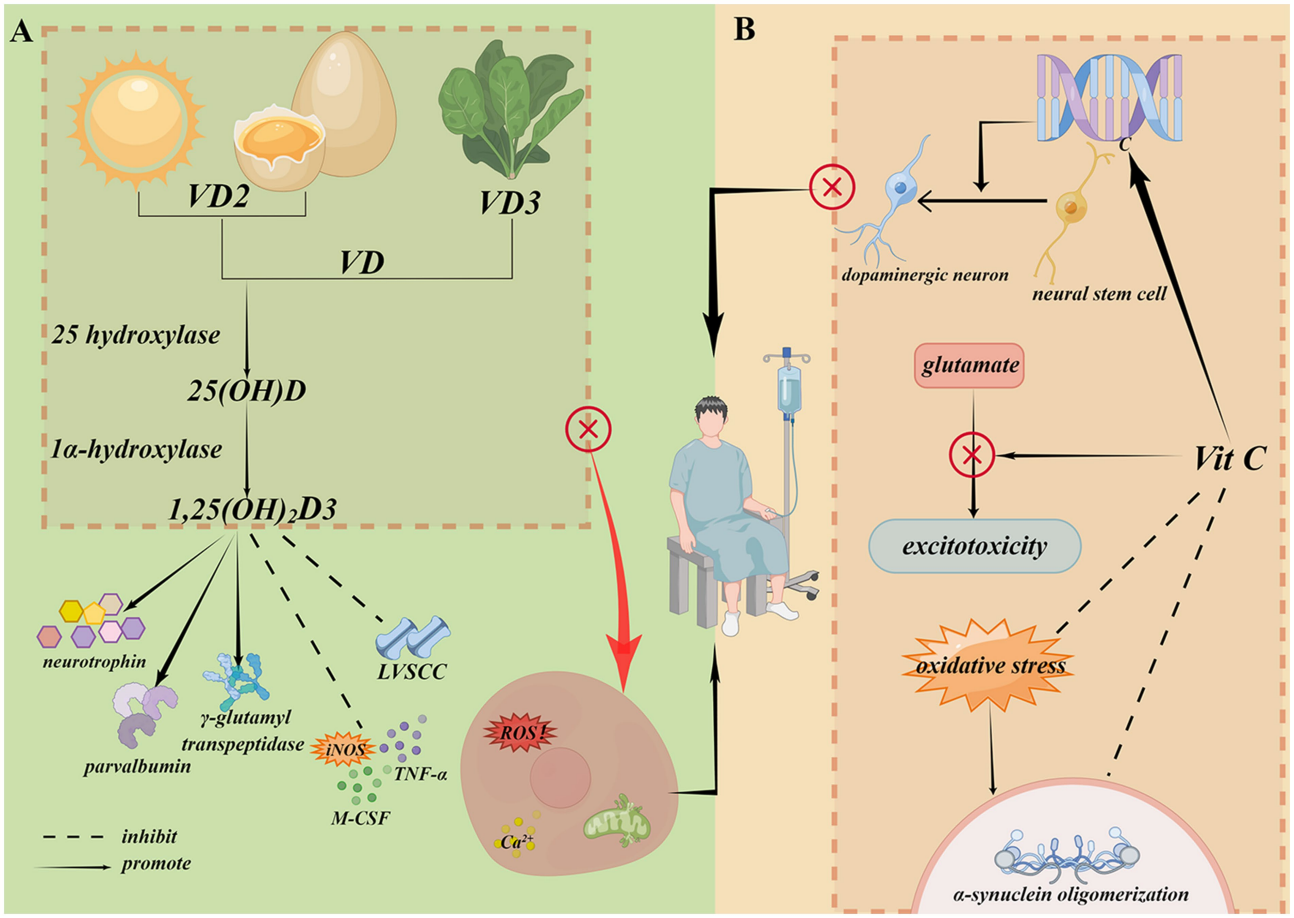
The neuroprotective effects of vitamin D and vitamin C. **(A)** Vitamin D3 is mainly derived from animal foods and endogenous synthetic, while vitamin D2 is mainly derived from plant foods. 1,25(OH)2D3 induces to release neurotrophin and synthesize Ca^2+^ binding proteins such as parvalbumin, inhibits to synthesize inducible iNOS, M-CSF and TNF-α, down-regulates LVSCC and up-regulates the γ-glutamyl transpeptidase activity. **(B)** Vitamin C increases to induce midbrain derived neural stem cell into dopaminergic neurons, reduces α-synuclein oligomerization and exerts a neuroprotection role through preventing glutamate-mediated excitotoxicity. Created with Figdraw (www.figdraw.com). M-CSF, macrophage colony-stimulating factor; LVSCC, L-type voltage sensitive Ca^2+^ channel.

Although the potential effects of VD on the prevention and treatment of PD are e not clear, there is much evidence associated with its neuroprotective effects by reducing excitotoxicity in PD. In addition, 1,25(OH)2D3 induces the release of neurotrophin and synthesis of Ca^2+^-binding proteins such as parvalbumin, inhibits the synthesis of inducible iNOS, macrophage colony-stimulating factor, TNF-α, down-regulates L-type voltage sensitive Ca^2+^ channel (LVSCC), and upregulates γ-glutamyl transpeptidase activity, which are also reported to be the potential neuroprotective mechanisms of VD in PD (Figure 2A) (91–93).

VD increases several neutrophins, such as nerve growth factor, glial cell-derived neurotrophic factor, brain-derived neurotrophic factor, neurotrophin-3, ciliary neurotrophic factor, low-affinity p75 neurotrophin receptor, and transforming growth factor-b2, and down-regulates neurotrophin 4. VD is closely related to intraneuronal Ca^2+^ homeostasis and glial cytosolic Ca^2+^ concentration, and plays a role in regulating LVSCC, up-regulating parvalbumin and calbindin synthesis, and reversing neuronal functions. VD deficiency results in neuronal cell death by inducing neuroexcitatory toxicity via triggering a sudden increase in cytoplasmic Ca^2+^, elevating ROS generation, and inducing mitochondrial dysfunction. VD also exerts antioxidant effects, reduces ROS generation, nitric oxide synthase synthesis, and NFkB activity, and enhances gamma-glutamyl transpeptidase activity. Therefore, VD deficiency might result in dopaminergic neuron loss in the brain, contributing to PD (94–96). The current research and investigation shows that both serum VD and dietary VD intake are low in patients with PD, and there is a strong correlation between lower serum VD and dietary VD intake and the severity of PD symptoms. Higher levels of both serum VD and dietary VD intake are protective against PD.

### 3.2 Antioxidant vitamins

Oxidative stress is a potential mechanism in the pathogenesis of PD, and antioxidants, such as vitamin C (Vit C), vitamin E, and vitamin A and their active derivatives, are thought to potentially protect cells from oxidative damage, thereby inhibiting the onset and progression of PD. Although many epidemiological studies have examined the relationship between dietary antioxidant intake and the risk of PD, the results have been highly inconsistent (97, 98).

Vit C has been suggested to exert a potential effect on the differentiation of dopaminergic neurons. It was revealed that Vit C increases to induce midbrain-derived neural stem cells into dopaminergic neurons by increasing 5-hydroxymethylcytosine and decreasing histone H3 lysine 27 tri-methylation generation in the dopamine phenotype gene promoters, which are catalyzed by ten-eleven-translocation 1 (Tet1) methylcytosine dioxygenase 1 and histone H3K27 demethylase (Jmjd3), respectively. Vit C also exerts these effects by regulating Tet1 and Jmjd3 activities as a co-factor, and also plays an indirect role in α-synuclein oligomerization. Oxidative stresses, including 4-hydroxy-2-nonenal, nitration, and oxidation, modify posttranslational α-synuclein to promote α-synuclein oligomerization, which inhibits oxidative stress responses as an antioxidant. Vit C also reduced Cu^2+^-mediated 3,4-dihydroxyphenylacetaldehyde-induced α-synuclein oligomerization in rat pheochromocytoma PC12 cells. In addition, Vit C exerts a neuroprotective effect by preventing glutamate-mediated excitotoxicity in PD. Vit C prevented the death of human dopaminergic neurons following glutamate neuroexcitotoxicity induced by stimulating α-amino-3-hydroxy-5-methyl-4-isoxazole propionic acid and metabotropic receptors, and N-methyl-d-aspartate and kainate receptors in *in vitro* study (Figure 2B) (99, 100).

Clinical studies have shown that Vit C deficiency among PD patients with PD is widespread, and intracellular Vit C seems to be associated with PD. Lymphocyte Vit C levels in patients with severe PD were significantly lower than those in patients with less severe PD. A few clinical studies have suggested that Vit C treatment plays a role in the course of PD, and that dietary vitamin C intake is significantly associated with reduced PD risk (99). However, the results of four prospective cohort and eight case–control studies showed that a high-Vit C diet did not significantly reduce the developmental risk of PD (101). Moreover, Vit C has the potential to increase L-dopa absorption in elderly patients with PD. An *in vitro* study revealed that Vit C is a strong inducer of L-dopa production in pre-grown mycelia of *Aspergillus oryzae* NRRL-1560 (99). Based on the current viewpoints, a high-Vit C diet is beneficial for preventing and reducing PD risk.

Vitamin E insufficiency is involved in a variety of neurodegenerative diseases such as PD. Although the effects of vitamin E in neurodegenerative diseases, including PD, are not completely understood, there are various potential mechanisms underlying the risk of PD, such as the neuroprotective effect of vitamin E against oxidative stress damage and the inhibition of the expression of many genes involved in neurodegeneration development. To date, several studies have investigated the association between vitamin E intake and vitamin E levels in body fluids and neurodegenerative diseases, including PD. Among these, vitamin E can play a neuroprotective role in neurodegeneration with respect to PD. Moreover, a vitamin E diet is also associated with risk factors for neurodegenerative diseases such as PD (102).

Retinoic acid is an important bioactive derivative of lipophilic vitamin A, and the alteration of retinoic acid signaling may participate in the pathogenesis and pathophysiology of PD. Vitamin A participates in several important homeostatic processes including cell differentiation, antioxidant activity, inflammation, and neuronal plasticity. The roles of vitamin A and its derivatives in the pathogenesis and pathophysiology, as well as their potential therapeutic effects in neurodegenerative diseases such as PD, have been studied by many researchers. Therefore, vitamin A may have some potential effects at the crossroads of multiple environmental and genetic factors in PD. These principal biological systems and mechanisms controlled by both vitamin A and its derivatives show that it could be exploited as a potential candidate therapeutic pharmaceutical in PD because of its potential effects on the survival of dopaminergic neurons, oxidative stress, neuroinflammation, circadian rhythms, homeostasis of the enteric nervous system, and hormonal systems. Aldehyde dehydrogenase 1A1 (ALDH1A1), an enzyme expressed by dopaminergic neurons for the detoxification of these neurons, is controlled by retinoic acid and plays a crucial role of aldehyde dehydrogenase enzymes in the survival of dopaminergic neurons by controlling the ALDH1A1 enzyme in PD (103). In view of the present information about the effects of vitamin E on the pathogenesis of PD, a vitamin E diet should also be associated with risk factors for PD.

## 4 Fatty acids

Fatty acids can be divided into saturated fatty acids (SFA), mainly from dairy products and meat, monounsaturated fatty acids, mainly from sunflower oil, peanut oil, olive oil, and polyunsaturated fatty acids, mainly from vegetable oils, fish oil, and marine animals, according to the saturation degree. Polyunsaturated fatty acids (PUFA) can be divided into n-3, n-6, and n-9 series, according to their double bond positions. The n-3 and n-6 series are closely related to the human health, the n-3 series include alpha-linolenic acid (ALA), eicosapentaenoic acid (EPA), docosahexaenoic acid (DHA), the n-6 series mainly include linoleic acid and arachidonic acid (AA) (104).

The levels of n-6 and n-3 polyunsaturated fatty acids in the cerebral cortex of PD patients were significantly lower, especially AA and DHA, however, SFA was found to be significantly higher in PD patients (105). Total n-3 unsaturated fatty acid levels and DHA concentrations significantly increased and total n-6 polyunsaturated fatty acids and AA levels significantly decreased in the motor cortex of the PD mice model heavily fed DHA, and both striatal dopamine levels and tyrosine hydroxylase immunoreactivity were observed to increase and increase, respectively. This suggests that feeding polyunsaturated fatty acids could partially rescue and repair the dopaminergic system (106). In addition, n-3 PUFAs attenuated nigrostriatal dopaminergic neuronal damage and alleviated motor impairment in a PD mouse model (107).

Many epidemiological studies in recent years have attempted to discover the relationship between dietary polyunsaturated fatty acids and the development of PD. A cohort study found that the intake of total fat, monounsaturated fatty acids, and polyunsaturated fatty acids was significantly associated with a lower risk of PD, however, no association was found between dietary saturated fat, cholesterol, or trans-fat and PD (104). In addition, a case–control study that provided dietary information also showed that a high intake of n-3 polyunsaturated fatty acids (≥0.847% energy supply ratio) compared to low intake (0–0.671% energy supply ratio) was negatively associated with PD (108). Another cohort study adjusting for the established risk factors for PD and the intake of major fatty acids only revealed a negative association between the dietary monounsaturated fatty acids and the risk of PD in women, with no statistically significant association between the dietary saturated fatty acids, n-3 and n-6 fatty acids, and the risk of PD (109).

Lipidomics provides some novel insights and answers regarding the role of lipids in PD pathology. Among the lipidomic studies, it has been demonstrated that α-synuclein binding is not only preferential to the specific lipid family but also to the specific molecular species of lipids. This suggests that these lipid-protein complexes enhance interactions with synaptic membranes, which affects the oligomerization and aggregation of α-synuclein, and interferes with the catalytic activity of cytoplasmic lipid enzymes and lysosomal lipases, thereby affecting lipid metabolism. A study on genetic linkages between aberrant lipid metabolism and PD directly demonstrates that glucocerebrosidase and sphingomyelin phosphodiesterase 1 mutations enhance PD risk in humans, and that galactosylceramide beta hydrolase function loss increases α-synuclein aggregation and accumulation in experimental murine models. Furthermore, many lipidomic studies have demonstrated that PD-specific lipids are significantly altered in the brains and plasma of patients with PD, such as alterations in the lipid composition of lipid rafts in the frontal cortex. Lipid dysregulation promotes the pathogenesis of PD by oxidative stress and inflammation, such as proinflammatory lipid mediators of platelet-activating factors, which play a key role in the progressive neurodegeneration associated with α-synuclein intracellular traffic in PD. In addition, many genetic risk factors for PD participate in normal lipid metabolism and function. Genes such as phospholipase A2 group VI and scavenger receptor class B member 2 are involved in glycerophospholipid and sphingolipid metabolism, which are either directly or indirectly related to the risk of PD (110). Although the importance and relevance of polyunsaturated fatty acids and PD show a positive trend in animal models, inconsistent results exist in the epidemiological association of PUFAs intake with PD risk, and further studies are needed. Therefore, dietary fatty acid intake and metabolism should not be ignored in the development and prevention of PD.

## 5 Dietary patterns

At present, a large number of studies have mainly focused on the association of single nutrients or specific food components with PD. However, the individual intake of nutrients is not isolated, and complex interactions usually exist among different dietary habits, nutrients, and foods. Therefore, the association between diet and PD has been explored from the perspective of overall dietary patterns, which may better reflect the influence of dietary factors on PD (7).

### 5.1 Mediterranean diet

**Figure 3:**
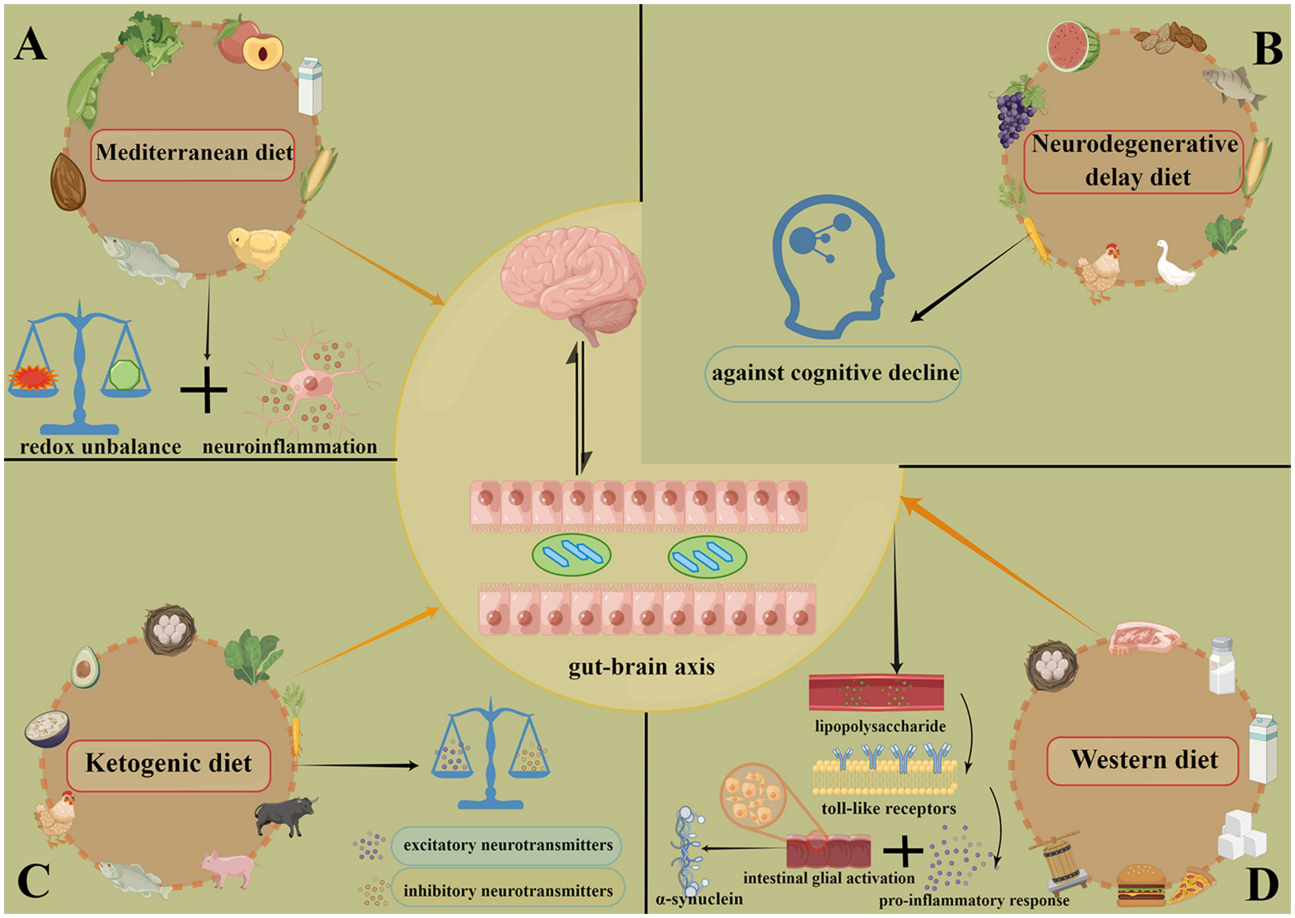
The composition of 4 diets and their effects in PD. **(A)** MeDiet is composed of abundant antioxidants, anti-inflammatory agents, minerals and vitamins. The main benefits of this diet are antioxidant and anti-inflammatory. **(B)** Neurodegenerative delay diet emphasizes plant-based foods, this diet may protect against the cognitive decline. **(C)** Ketogenic diet mainly reduces a glucose supply and promotes ketogenesis, exerts the neuroprotective effects by controlling and moderating the balance between excitatory and inhibitory neurotransmitters. **(D)** Western diet is mainly composed of high calories, high fat and high protein foods, it affects the microbiota-gut-brain axis and promotes the development of PD. In contrast, MeDiet and Ketogenic diet exert the neuroprotective effects by altering the composition of gut microbiome. Created with Figdraw (www.figdraw.com). MeDiet, Mediterranean diet; PD, Parkinson’s disease.

The traditional Mediterranean diet (MeDiet), recognized by the United Nations Educational, Scientific and Cultural Organization as an “Intangible Cultural Heritage of Humanity,” is characterized by a complete, minimally processed plant-based diet (cereals, legumes, vegetables, fruits, and nuts), small amounts of meat, milk, and dairy products, and moderate amounts of red wine, nuts, olive oil, and fatty fish (Figure 3A) (111, 112). At present, special attention has been paid to the potential relationship between Mediterranean diet (MeDiet) and PD risk.

The MeDiet is composed of abundant antioxidants, anti-inflammatory agents, minerals, and vitamins. Although the roles of the MeDiet in PD have not been comprehensively studied and the investigation results are sometimes contradictory, collectively, the MeDiet has been shown to exert a neuroprotective effect in the onset and progression of PD in the current study. One of the main beneficial properties of the MeDiet is its ability to effectively regulate both redox imbalance and inflammatory response. Because neuroinflammation and oxidative stress are recognized as two important factors participating in the onset and development of PD, the beneficial roles of the MeDiet could rely on the antioxidant and anti-inflammatory properties of this dietary pattern. Among the constituent elements of MeDiet, vitamins, omega-3 polyunsaturated fatty acids (ω3-PUFAs), and polyphenols have been found to have protective properties against PD in largely studies (113).

PUFAs are also the major elements of MeDiet, 3 principal elements of ω3-PUFAs consist of EPA, docosahexaenoic acid (DHA, typically from fish oil), and alpha-linolenic acid (ALA, typically from plant sources). Ω3-PUFAs are important constituents of membrane phospholipids and exert an important effect on modulating the physicochemical properties of membranes, which in turn affects cellular functions. Many aspects of MeDiet ω3-PUFAs, including their antioxidant and anti-inflammatory properties and various mechanisms of action behind their anti-inflammatory activity, are beneficial in the prevention and/or treatment of PD (7, 114, 115).

One of the major MeDiet features is the abundance of olive oil in the composition of foods. Olive oil, which is abundant in polyphenols, partly exerts the benefit of the MeDiet dietary pattern. The neuroprotective roles associated with the daily consumption of olive oil are mostly related to the antioxidant and anti-inflammatory effects of polyphenolic fractions in the MeDiet. In addition to the current mechanism of the effects of the MeDiet on the response to oxidation and inflammation, another candidate and potential neuroprotective mechanism might be associated with the roles mediated by this dietary pattern in regulating and/or protecting the gut microbiota composition of the gut-brain axis (7, 114).

In a cohort study of a longitudinal survey of aging and diet, it was found that the probability of PD was lower in the group with higher adherence to the MeDiet, which suggests that adherence to the MeDiet is associated with a lower likelihood of PD in older adults (116). Moreover, in a female population-based cohort study, adherence to MeDiet in midlife was negatively associated with the development of PD in later life (117). Therefore, the potential correlation between MeDiet and the risk of PD needs to be further investigated and studied to clarify the effectiveness of the MeDiet dietary pattern on the effects of PD and its complications.

### 5.2 Neurodegenerative delay diet

The dietary approaches to stop hypertension diet (DASH) emphasizes fruits, vegetables and low-fat dairy products, including whole grains, poultry, fish and nuts, and reduces fat, red meat, candy and sugary drinks (Figure 3B). A Mediterranean-DASH intervention for neurodegenerative delay (MIND) diet developed specifically to promote brain health is a combination of Mediterranean and DASH diets, with many of the same components, but with an emphasis on natural, plant-based foods, especially increases in the consumption of berries and leafy greens, and limits the intake of animal and high-saturated fat foods (118).

In a cross-sectional study with MIND diet adherence scores, it was found that MIND diet adherence was significantly associated with a later age of PD onset, especially in women (119). In a cohort study, a structured clinical assessment of PD symptoms and progression rates combined with different dietary pattern scores and analyzed using the Cox proportional-hazards model revealed that each 1-point increase in MIND score was associated with a 13% reduction in PD incidence, and that higher MIND dietary compliance was associated with slower progression of PD (120).

### 5.3 Ketogenic diets

A ketogenic diet (KD) is a diet high in fat, low in carbohydrates, and adequate in protein (Figure 3C), which results in a reduction in glucose supply and enhancement of ketogenesis (121). KD is effective in the treatment of refractory epilepsy and improves non-motor symptoms of PD.

KD appears to provide a potential therapeutic benefit for patients with neurodegenerative diseases, including PD, by controlling and moderating the balance between excitatory and inhibitory neurotransmitters, as well as modulating inflammation or altering the composition of the gut microbiome (121, 122). In a random study of PD patients in the KD group and a low-fat, high-carbohydrate diet group, the results showed that both diet groups showed significant improvements in motor and non-motor symptoms, however, the KD group showed greater improvements in non-motor symptoms, including cognitive function (123). Moreover, the KD effects of low carbohydrates improve executive performance and memory in participants with mild cognitive impairment associated with PD (124).

Although the current studies on KD and PD show some promising results, there are fewer intervention studies in PD populations, relatively short treatment durations in those with small sample sizes, and the lack of PD-specific outcomes that do exist. Thus, research on the effects of KD therapy in PD is still at a preliminary stage. In addition, long-term adherence to a KD can lead to loss of appetite, gastrointestinal discomfort, and other side effects (125), however, the elderly often suffer from possible concomitant diseases such as diabetes and malnutrition, which affects compliance and limits the application of KD (126). At present, it is difficult to determine the importance of this diet in the treatment of PD, and more research is necessary to recommend or refute the use of KD therapy in PD.

### 5.4 Western diet

The dietary structure of western diet pattern represented by western developed countries is characterized by high energy food intake, high protein, saturated fat, refined cereals, sugar, alcohol and salt, and low intake of omega-3 fatty acids, fruits and vegetables, thus has the diet pattern of “three highs” of high calories, high fat and high protein. Recent epidemiological studies have shown that a long-term Western diet affects the microbiota-gut-brain axis and promotes the development of neurodegeneration, leading to the development of PD (Figure 3D) (127, 128).

It has been proposed that the western diet-induced intestinal ecological dysregulation and the altered intestinal barrier function can induce neuroinflammation, in turn can activate Toll-like receptors, then cause a proinflammatory response and the intestinal glial activation, ultimately contributes to the α-synuclein pathology in the pathogenesis of PD (129). Several foods in the Western diet, such as beef, ice cream, fried foods, and cheese, are closely associated with the rapid progression of PD (130). Ultra-processed foods are also a hallmark of the Western diet and are thought to trigger low-level systemic inflammation and oxidative changes that favor the development of neurodegenerative diseases, including PD (131). In general, this dietary behavior not only leads to an increased incidence of cardiovascular and metabolic diseases but also increases the risk of PD.

## 6 Conclusion

Although a growing number of clinical and experimental investigations have demonstrated the potential role of diet in the prevention of PD, the effects of dietary interventions in PD remain controversial. The components and pattern of diets not only reduce and prevent the generation of α-synuclein by intervening in oxidative stress and inflammation but also play a role in regulating the development, growth, and survival of dopaminergic neurons, including the balance regulation of the microbiota-gut-brain axis. Overall, plant-based, low-fat, high-fiber diets with antioxidant-rich dietary interventions can prevent and reduce the development, progression, and complications of PD and improve its clinical symptoms and signs. In the future, clinical studies on diet and PD need to conduct more large-scale long-term cohort studies and randomized controlled trials to explore the relationship between diet and PD in greater depth and provide more scientific evidence for proposing PD-related diet intervention strategies and preventing and treating PD using nonpharmacological means.
